# Supplementary material for: Jasmonate and Ethylene-Regulated Ethylene Response Factor 22 Promotes Lanolin-Induced Anthocyanin Biosynthesis in ‘Zaosu’ Pear (Pyrus bretschneideri Rehd.) Fruit
Source: Biomolecules. 2020 Feb 11;10(2):278. doi: 10.3390/biom10020278 (PMC7072184; doi:10.3390/biom10020278)
Supplement: Supplementary file 1 [file biomolecules-10-00278-s001.zip › biomolecules-699886 revised 3 supplementary original/Supplementary materials/EdanzEditing Certificate.pdf]

理文编辑

Certificate of Editing

Edited provisional title

Jasmonate- and ethylene-regulated ethylene response factor 22 promotes lanolin-induced anthocyanin biosynthesis in ‘Zaosu’ pear (Pyrus bretschneideri Rehd.) fruit

Client name and institution

Ting Wu, College of Horticulture, Northwest A&F University, Taicheng Road NO.3, Yangling 712100, Shaanxi Province, China

Date Completed
2020-02-06

Identification code
75249

Certificate issued by
Koji Yamashita
Managing Director and CEO

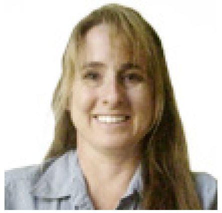

Expert Editor: Lesley Benyon
1999 PhD Genetics and Molecular Biology
University of North Carolina
Biochemistry and Cell Biology, Genetics, Plant Biology

www.liwenbianji.cn

While this certificate confirms the authors have used Edanz's editing services, we cannot guarantee that additional changes have not been made after our edits.
