# Supplementary material for: Jasmonate and Ethylene-Regulated Ethylene Response Factor 22 Promotes Lanolin-Induced Anthocyanin Biosynthesis in ‘Zaosu’ Pear (Pyrus bretschneideri Rehd.) Fruit
Source: Biomolecules. 2020 Feb 11;10(2):278. doi: 10.3390/biom10020278 (PMC7072184; doi:10.3390/biom10020278)
Supplement: Supplementary file 1 [file biomolecules-10-00278-s001.zip › biomolecules-699886 revised 3 supplementary original/Supplementary materials/Supplementary material.pdf]

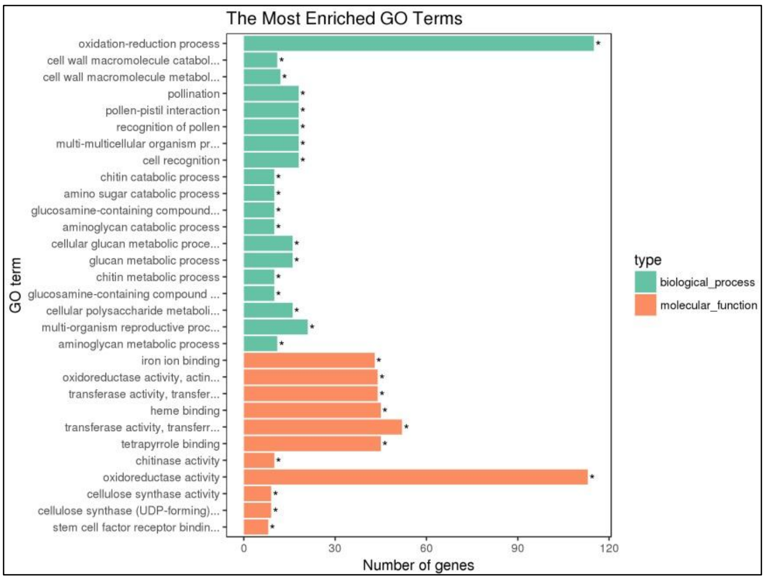

Figure S1. Gene ontology (GO) annotation and enrichment analysis of 748 DEGs.

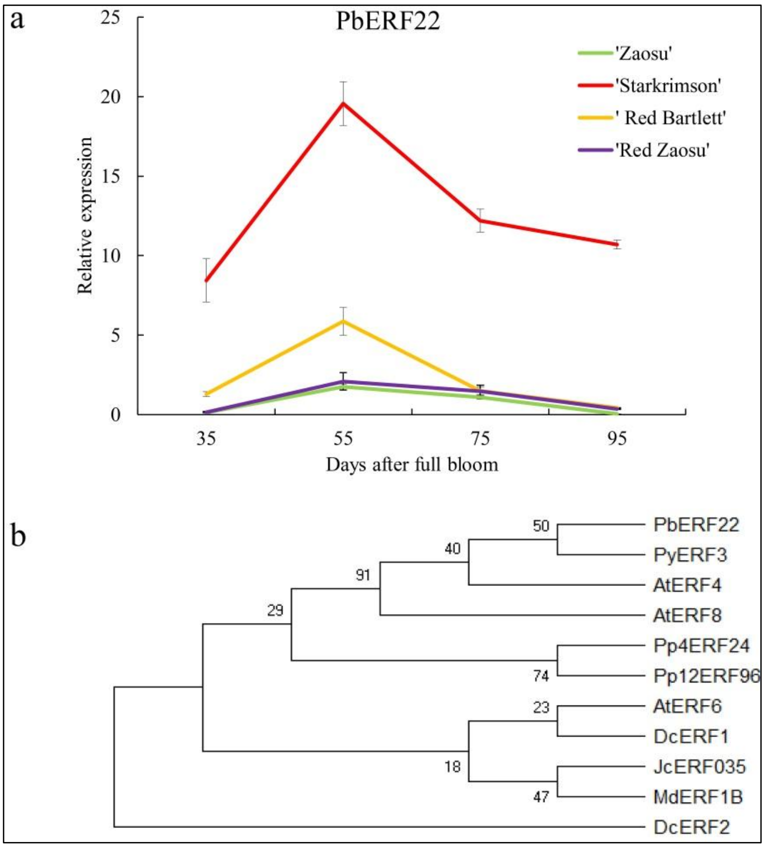

Figure S2. The expression levels of *PbERF22* in several red-skinned pear fruit, and Phylogenetic tree of ERFs from different species. (a) The expression of *PbERF22* in 'Zaosu', 'Starkrimson', 'Red Bartlett' and 'Red Zaosu' pear peel during fruit development. (**b**) Phylogenetic tree of previously reported ERF genes which associated with anthocyanin accumulation in different plant species. At, *Arabidopsis thaliana* (AAN12993.1, NP_567529.1, NP_188139.1, NP_175725.1); Jc, *Jatropha curcas* (XP_012093083.1); Md, *Malus domestica* (XP_008368126.2); Dc, *Daucus carota* (BAF75651.1, BAF75652.1); Pb, *Pyrus bretschneideri* (XP_009333695.1, XP_018505750.1, XP_009357258.1, ASY06613.1, XP_009357206.1).

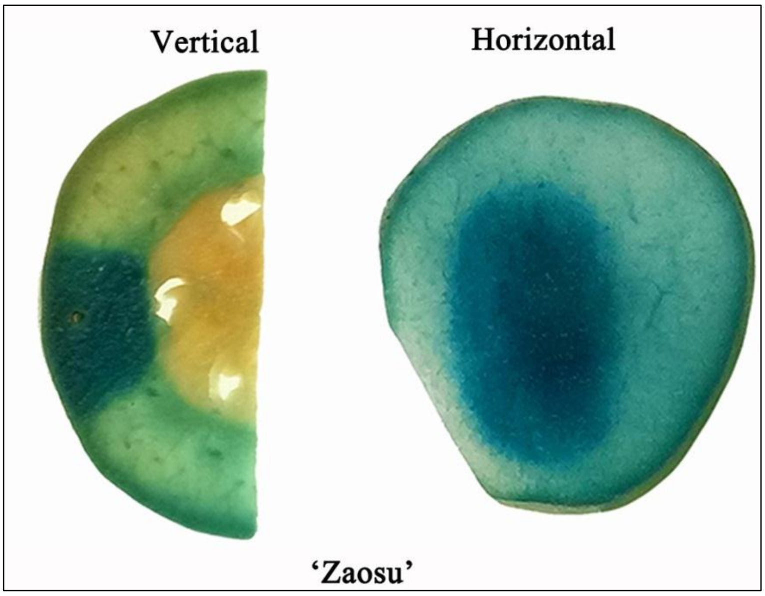

Figure S3. The GUS-staining of ‘Zaosu’ fruit infiltrated with pGreenII 62-SK-GUS shown in vertical and horizontal planes.

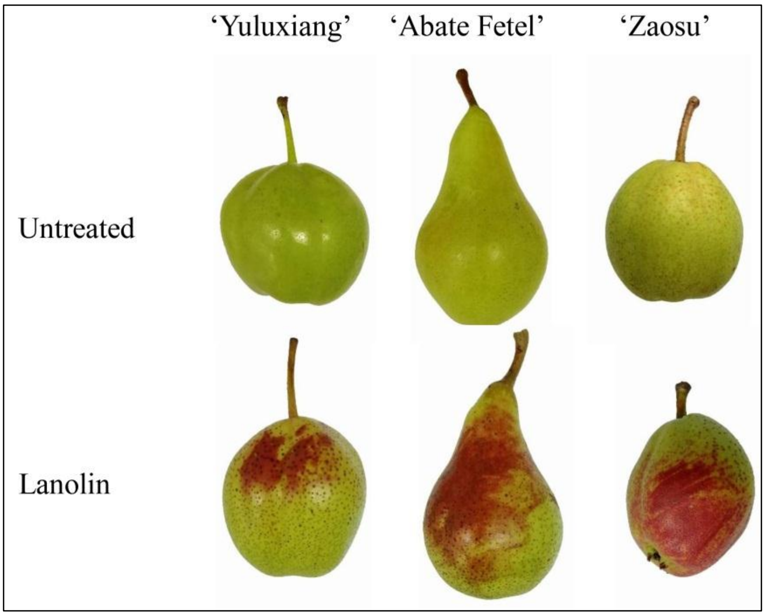

Figure S4. Phenotypes of several untreated and lanolin-treated green-skinned pears at the same developmental stages.

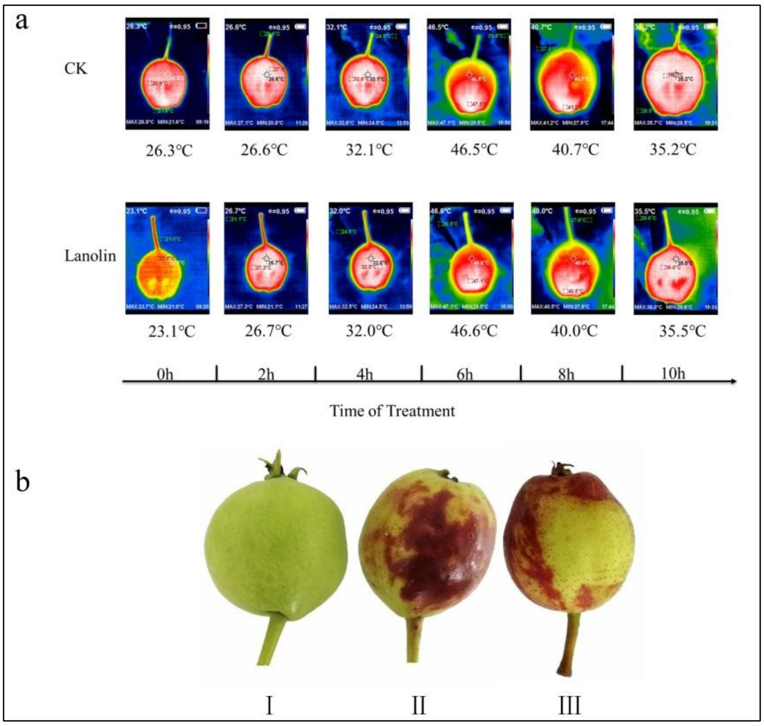

Figure S5. 'Zaosu' fruit surface thermo gram and the lanolin's permeability analysis. (**a**) 'Zaosu' pear fruit were harvested at 100 DAFB, treated with lanolin (Lanolin) and water (CK), the thermogram of fruit surface at different time points during normal light. (**b**) 'Zaosu' pear fruit at 60 DAFB were treated with control (Ⅰ), lanolin (Ⅱ), and plastic wrap covered with lanolin (Ⅲ).

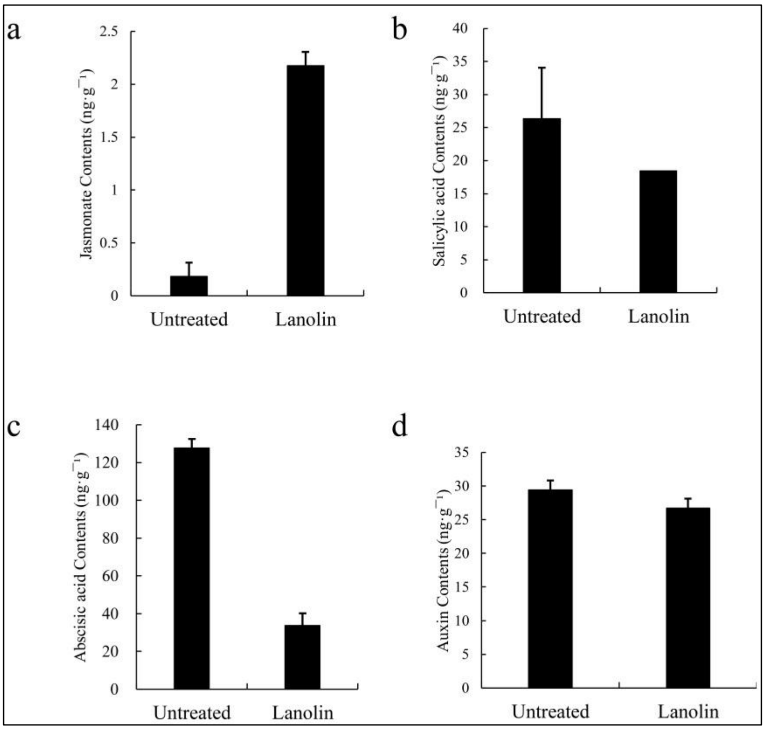

Figure S6: Quantifications of phytohormones. HPLC detected the phytohormones contents of jasmonate (**a**), salicylic acid (**b**), abscisic acid (**c**) and auxin (**d**) in 'Zaosu' fruits peel on 10 d during untreated and lanolin treatment, respectively.

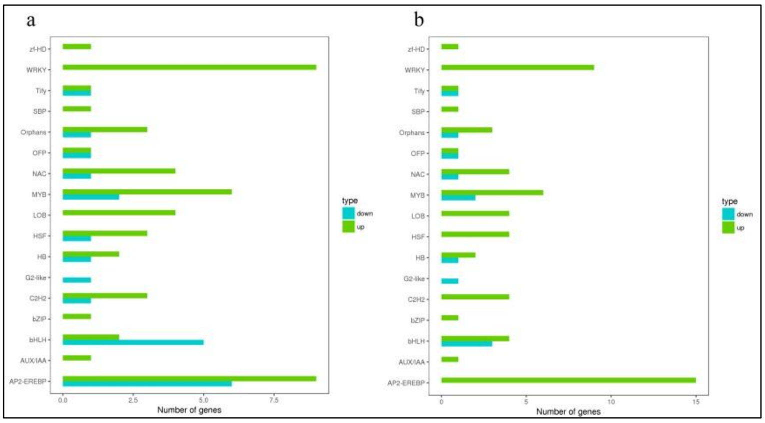

Figure S7. Transcription factor family analysis at 10 (a) and 20 (b) d of DEGs in lanolin-treated and untreated samples. The numbers of up-regulated genes and down-regulated genes comparing to control were showed with green (up) and blue (down), respectively.

Table S1. List of primers for qRT-PCR

Gene	Forward primer (5'to 3')	Reverse primer (5'to 3')
PbERF22	TCCGCGAACCTGGAAAGAAA	GTCCTTTGAGGTGCAATGCC
PbACS1	ATCTCGTTTTGGACGTGCTC	ACGACAGCTTTCCTATTTAGTCCAT
PbACO1	CGCGGGGACTCAACAAAAA	GGTTGCTACTCTCTCTTCACCA
PbAOS	TAAGTCGCAAAAGCATGCACA	GGTGAGATTTTGATTTGGAGTTTGG
PbAOC	CGTTCAAGCCCTGCGTATCT	CGCCGTTATCCCCAATCGT
PbLOX	AGCAGCAAGATGGGGAGAGT	ATGCACACGATCAAGAACCG
PbOPR3	CTCTCACAGGGTGGTGCTG	CACTGATTAGAAAGCCGCCG
PbCHS	ACCCAGTGCCCGAAGTCG	AGCGAGTTCCAGTCTGAAATGC
PbDFR	GGACGACCTCTGCCTTTCTCAC	GAACTCAAACCCTATCTCCCTCAAC
PbANS	CTGGGCAGCTTGAGTGGGAG	CCACCAACTTCTTTCTCCAGCC
PbUFGT	GTGGAGGACGTGTTGGAGATAGG	CCTCGCACGATGCTTTATTGG
PbMYB10	GAGACCTTGTTCGAAGGCGA	TTGCCGACAGTCGATCATCA
PbMYB10b	ATACAGACAATGTTGATGGAACACC	CGATTGCTTGAGACTTTGGACC
PbbHLH3	TAATCGAGAGTGATGGGCTGTTG	TCTTGCCACTCACGTTATCCTTC
PbACTIN	TGAGTCACACTGTGCCAATCTATG	TGGTGAACATGTACCCTCTTTCAG

Table S2. List of primers for constructing vectors

Gene	Forward primer (5'to 3')	Reverse primer (5'to 3')
PbERF22-pGreen II 62-SK	TAGAACTAGTGGATCCATGGA ATCCCAGGCTGCAG	CGGTATCGATAAGCTTCTAGGG GTCCCAAATGGACC
PbMYB10-pGreen II 62-SK	TAGAACTAGTGGATCCATGGA GGGATATAACGTTAACTTGA	CGGTATCGATAAGCTTACTATT CTTCTTTTGAATGATTCCAAA
PbMYB10b-pGreen II 62-SK	TAGAACTAGTGGATCCATGAT GAGGAAGGGTGCCTG	CGGTATCGATAAGCTTCTAAAT CTTAGTTATCTCTTCTTCTAG
PbbHLH3-pGreen II 62-SK	TAGAACTAGTGGATCCATGGC TCAGAACCATGAGAGG	CGGTATCGATAAGCTTGCACTT ACCAGCAATTTTCC
PbUFGT pro-LUC	CGGTATCGATAAGCTTGTGCC TCCCGTAGGGCTAC	TAGAACTAGTGGATCCCGGCGG TGCCATTACAAC
